# Heterologous production of levopimaric acid in *Saccharomyces cerevisiae*

**DOI:** 10.1186/s12934-018-0964-1

**Published:** 2018-07-18

**Authors:** Ting Liu, Chuanbo Zhang, Wenyu Lu

**Affiliations:** 1 School of Chemical Engineering and Technology, Tianjin University, Tianjin, 300072 People’s Republic of China; 2 Key Laboratory of System Bioengineering, Tianjin University, Ministry of Education, Tianjin, 300072 People’s Republic of China; 3 Collaborative Innovation Center of Chemical Science and Engineering (Tianjin), SynBio Res Platform, Tianjin, 300072 People’s Republic of China

**Keywords:** *Saccharomyces cerevisiae*, Levopimaradiene, Levopimaric acid, Synthetic biology

## Abstract

**Background:**

Levopimaric acid (LA), a type of diterpene resin acid produced by plants, is a significant industrial intermediate that is mainly produced via phytoextraction. This work aimed to apply synthetic biology to produce LA in yeast strains from a simple carbon source.

**Results:**

Levopimaradiene (LP), the precursor of LA, was produced via LP synthase (LPS) expression in yeast. LPS was then modified by N-terminal truncating and site-directed mutagenesis. The strain containing t79LPS^MM^ (79 N-terminal amino acid truncating and M593I/Y700F mutation) produced 6.92 mg/L of LP, which were 23-fold higher than the strain containing LPS. Next, t79LPS^MM^ was expressed in a new metabolically engineered chassis, and the final LP production increased 164-folds to 49.21 mg/L. Three cytochrome P450 reductases (CPRs) were co-expressed with CYP720B1 (the enzyme responsible for LA production from LP) in yeast to evaluate their LA producing abilities, and the CPR from *Taxus cuspidata* (TcCPR) was found to be the best (achieved 23.13 mg/L of LA production). *CYP720B1* and *TcCPR* genes overexpression in the multi-copy site of the *S.cerevisiae* genome led to a 1.9-fold increase in LA production to 45.24 mg/L in a shake-flask culture. Finally, LA production was improved to 400.31 mg/L via fed-batch fermentation in a 5-L bioreactor.

**Conclusions:**

This is the first report to produce LA in a yeast cell factory and the highest titer of LA is achieved.

**Electronic supplementary material:**

The online version of this article (10.1186/s12934-018-0964-1) contains supplementary material, which is available to authorized users.

## Background

Terpenes represent a large class of natural secondary metabolites and have attracted industrial interest in cosmetics, pharmaceuticals, and potential biofuels [1, 2]. Diterpenes, types of compounds with 20 carbon atoms in their skeleton, are important plants metabolites that are used to defend insects or pathogens [3]. Some plant origin diterpenes are even found to have industrial applications, such as taxol mainly from *Taxus brevifolia* [4], tanshinones [5] from *Salvia miltiorrhizha*, and ambroxan from *Salvia sclarea* [6]. LA, an important diterpene resin acid in conifers, is a significant industrial intermediate; its Diels–Alder reaction products are widely used in coatings, printing inks, plasticizers and adhesives [7, 8]. However, these high value-added terpenoids rarely accumulate in their native host and are difficult to chemically synthesize due to their complicated chemical structure.

In recent years, synthetic biology has developed ways to synthesize these compounds in heterologous hosts. Two chassis, *Escherichia coli* and *Saccharomyces cerevisiae*, are often selected for these process [9, 10]. In *E.coli*, terpenoids are produced via the 2-C-methyl-d-erythritol-4-phosphate (MEP) pathway. LP, the precursor of LA, was produced via a precursor pathway metabolically engineered *E.coli*; the levopimaradiene synthase (LPS) from *Ginkgo biloba* and geranylgeranyl diphosphate synthase (GGPPS) from *Taxus canadensis* were designed by protein engineering, and the combination of these two strategies increased levopimaradiene production about 2600-fold to 700 mg/L in a benchscale bioreactor [11]. Although great progress has been made in engineering *E.coli* to produce diterpenes, many problems still hinder its industrial application. For example, cytochrome P450 monooxygenase (P450 s) from plants often loses function when it is expressed in *E.coli*. Comparatively, *S. cerevisiae* provides a more suitable environment for the functional expression of cytochrome P450 s and other downstream pathway enzymes [12, 13]. Therefore, owing to its robustness and compatibility, *S. cerevisiae* is the favorable host that is often chosen to produce isoprenoids.

In *S. cerevisiae*, the common precursors for isoprenoid production, isopentenyl diphosphate (IPP) and dimethylallyl diphosphate (DMAPP), are synthesized from acetyl-COA and then are condensed to form geranyl diphosphate (GPP) and farnesyl diphosphate (FPP) by farnesyl diphosphate synthase (ERG20). Geranylgeranyl diphosphate (GGPP), the precursor of diterpene, is synthesized by geranylgeranyl diphosphate synthase (BTS1) with FPP and IPP [14]. Engineering the structural genes of the mevalonate pathway (MVA) for terpene precursors supply was the easiest and most widely applied strategy [10]. BTS1p is the first enzyme that controls the flux toward diterpene production and competes for FPP with squalene (SQ) synthase in *S.cerevisiae* [15]. SQ is the crucial intermediate in sterol synthesis that is essential for the growth of yeast and much effort has been made to balance the native and heterologous pathway on this node [16, 17]. In order to enhance the efficiency of BTS1, heterologous GGPPS from plants or the BTS1 and ERG20 fusion protein was often employed [18]. Native ERG20 was reportedly engineered to produce GGPP and the diterpene sclareol production was significantly improved by using a combination of protein and genetic engineering strategies [6].

**Fig. 1:**
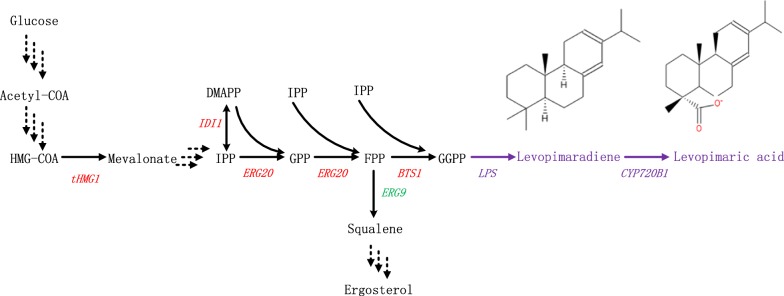
Description of diterpene biosynthesis in *S.cerevisiae* containing native MVA and heterologous LA production pathways. Up-regulated and down-regulated genes are indicated in red and green, respectively. The heterologous pathway is indicated in purple

In conifers, diterpene resin acids are important secondary metabolites that are synthesized by diterpene synthases and cytochrome P450 s, especially the CYP720B family [19, 20]. LA is a type of diterpene resin acids which is carboxylated at the C-18 position of LP by P450 s via a three-step oxidation reaction with alcohol and aldehyde intermediates. Here, we constructed the LA biosynthesis pathway in *S. cerevisiae* and successfully produced LA (Fig. 1). With the modification of LPS, optimization of CYP720B1 and CPR co-expression, and MVA pathway regulation, the production of LA increased to 49.21 mg/L. Finally, the production of LA was further improved to 400.31 mg/L via fed-batch fermentation in a 5 L bioreactor.

## Results and discussion

### Levopimaradiene production in *S.cerevisiae*

**Fig. 2:**
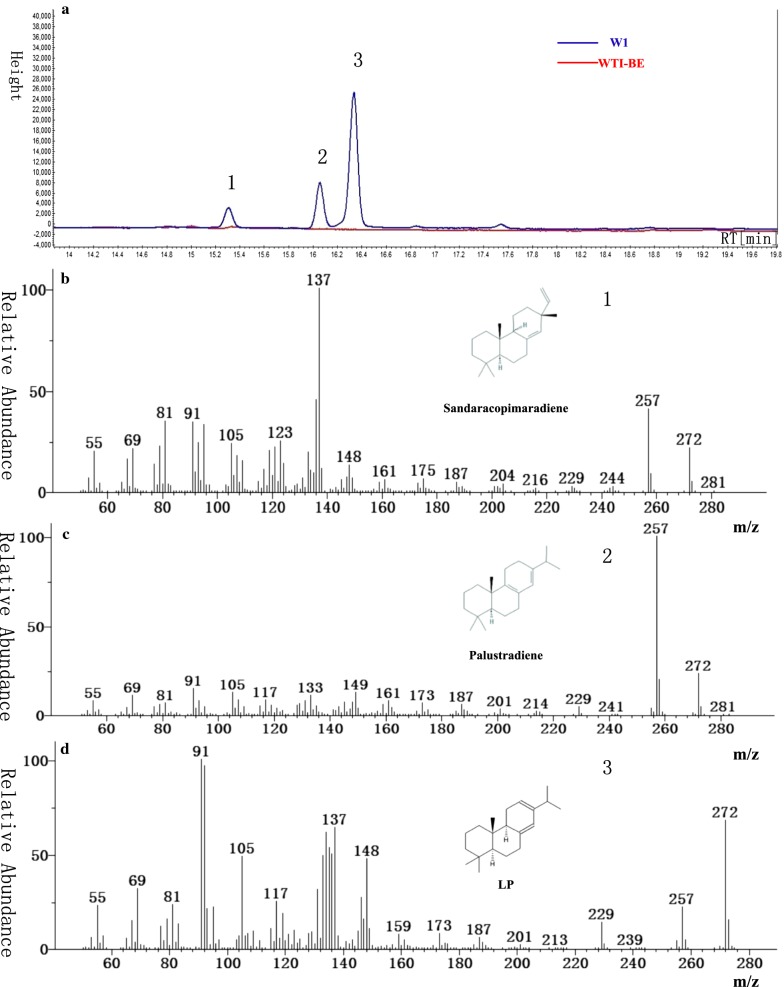
Levopimaradiene production in *S.cerevisiae.* **a** The chromatogram of the LP production strain. **b** GC–MS spectra of the product peak corresponding to sandaracopimaradiene (Peak 1). **c** GC–MS spectra of the product peak corresponding to palustradiene (Peak 2). **d** GC–MS spectra of the product peaks corresponding to LP (Peak 3). All product peaks were identified as previously reported in literatures [11, 21, 22]

Codon-optimized *GGPPS* and *LPS* gene expression in *E. coli* have been reported to only produce a small amount of LP [11]. Here, we first constructed WTI, an MVA pathway enhanced chassis, by over-expressing *tHMG1* and *IDI1* in the original W303-1a strain. BTS1p and ERG20p were then fused with the linker, GGGS, (BTS1-GGGS-ERG20p) and over-expressed in WTI, resulting in the WTI-BE stain. Codon-optimized *LPS* expression in WTI-BE formed W1. LP production by W1 was determined using gas chromatography-mass spectrometry (GC–MS) [21], and the titer was calculated to be 0.32 mg/L after 5 days of cultivation in shake-flasks (Fig. 2).

### LPS modification to improve levopimaradiene productivity

**Fig. 3:**
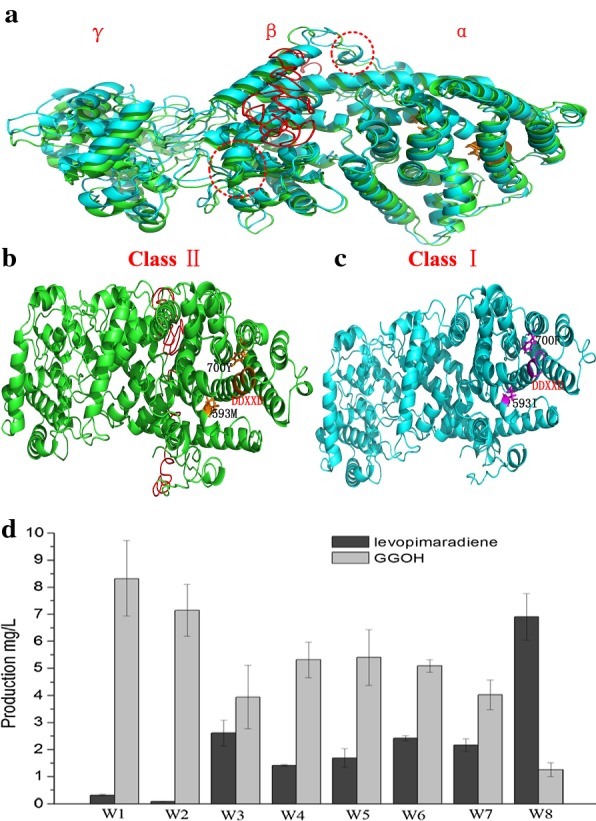
LPS modification improves LP production. **a** Homology modeling comparison between LPS (green) and T79LPS (blue); conserved DDXXD motif is marked in orange and the N-terminal plastid transit peptide is shown in red; circles in dotted lines show the differences between them. **b** Homology modeling of LPS; N-terminal 79 amino acid is shown in red; Met593 and Tyr700, located in the α domain binding pocket, are shown in orange; the conserved DDXXD motif is also in orange. **c** Homology modeling of T79LPS^MM^; Ile593 and Phe700, located in the α domain binding pocket, are shown in pink; the conserved DDXXD motif is also in pink. **d** LP and GGOH production by different strains with various LPS modifications: W1 (LPS), W2 (LPS^M593I/Y700F^), W3 (T40LPS), W4 (T40LPS^M593I/Y700F^), W5 (T60LPS), W6 (T60 LPS^M593I/Y700F^), W7 (T79LPS), and W8 (T79 LPS^M593I/Y700F^); Truncation is abbreviated to “T” and the number refers to the amino acid removed from the N-terminal. Error bars represent the standard deviation from three independent experiments

According to Ohto [15], geranylgeraniol (GGOH) accumulated when HMG1 and the ERG20-BTS1 fusion protein were co-expressed. GGOH is a by-product produced from GGPP via catalysis of a non-specific phosphatase. Hence, the GGOH was quantified and 8.51 mg/L of GGOH was produced by the W1 strain(Fig. 3d). However, the strain W1 produced only 0.32 mg/L of LP, which indicated that the LPS activity probably became the rate-limiting step in LP production. An LPS model was constructed by Leonard [11], and two important binding pockets (M593 and Y700) were found to have a significant influence on the LPS catalytic activity; when the M593I/Y700F modification was applied, diterpenoid production increased approximately tenfold. When *LPS* was cloned from the *Ginkgo biloba* cDNA library, a putative N-terminal plastid transit peptide was predicted; consequently, LP production increased fourfold with the removal of the 60 or 79 N-terminal residues in *E.coli* [21].

To intuitively understand LPS, its 3D structure (Fig. 3a–c) was predicted using homology modeling. Abietadiene synthase from *Abies grandis* was used as the template (PDB: 3s9v) [23]. As a bi-functional diterpene synthase (Fig. 3a), LPS is composed of two mono-functional modules: Class I and Class II. The Class II module consisting of a β and γ domain, catalyzes GGPP to form bicyclic prenyl diphosphate (CPP), whereas Class I module, consisting of an α domain, catalyzes CPP to form LP [24, 25]. The N-terminal plastid transit peptide also probably influences LPS activity. As shown in Fig. 3a, removal of the N-terminal plastid transit peptide changed the spatial structure of T79LPS (blue) when compared to its primary structure (LPS shown in green). The primary structure of LPS is shown in Fig. 3b and the conserved DDXXD motif is marked in orange. Double mutations (M593I/Y700F) in T79LPS^MM^ altered a part of the DDXXD motif from a helical to a loop structure (Fig. 3c). Both, N-terminal plastid transit peptide truncation and binding pocket (M593/Y700) mutation, affect the spatial structure and catalytic activity of LPS. Therefore, the synthetic effects were studied. As shown in Fig. 3d, removal of the 40, 60 and 79 N-terminal residues coupled with double mutations (M593I/Y700F) were tested for LP production and GGOH accumulation. All strains that contained LPS and only had their N-terminal residues removed performed better than the full-length ones. The best one, W3, which had 40 amino acids truncated, increased the production of LP by 8.6-fold to 2.63 mg/L. However, the result was different, when it combined with the double mutation. The W8 strain, which contained T79LPS^M593I/Y700F^, increased the LP production by 23-fold to 6.92 mg/L. GGOH production in W8 was 1.25 mg/L, which decreased 6.8-fold when compared to the original W1 strain. However, our results contradicted the findings of Leonard [11], who reported that the 40-N-terminal amino acids truncation was more stable than the 60- and 80-N-terminal amino acid truncation for LP production in *E.coli*. This discrepancy may be due to the different intracellular environments in *E.coli* and *S.cerevisiae.*

### Co-expression of CYP720B1 and CPR

**Fig. 4:**
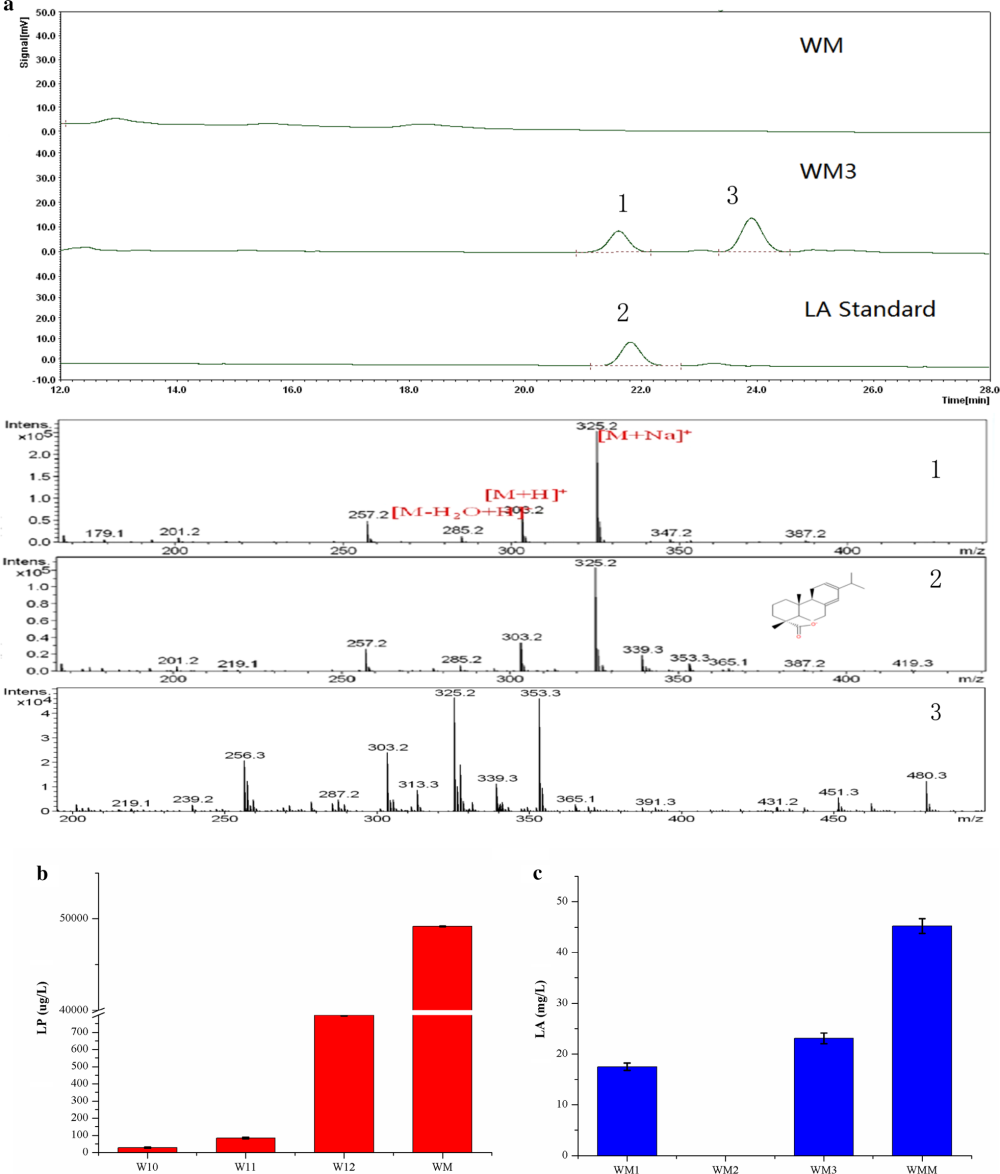
LA production in a high LP accumulation strain WM. **a** Liquid chromatography-mass spectrometry (LC–MS) analysis of the diterpenes produced by WM and WM3: 1, GC/MS of biosynthetic LA; 2, GC/MS of LA standard; 3, GC/MS of unknown peak produced by CYP720B1. **b** LP production in different metabolically engineered yeast strains. **c** LA production in the different *CYP720B1* and *CPR* co-expression strains, WM1 (*CYP720B1*-*AtCRP1*), WM2 (*CYP720B1*-*AtCRP2*), and WM3 (*CYP720B1*- *TcCPR*). Error bars represent the standard deviation from three independent experiments

LA biosynthesis begins with cyclization of GGPP by LPS, which was cloned and identified from *Ginkgo biloba* [21]. Two MVA pathway enhanced chassis, WTI (*tHMG1* and *IDI1* were overexpressed with the *P*_*PGK1*_ and *P*_*TDH3*_ promoters respectively) and WTE (*tHMG1* and *ERG20* were overexpressed with the *P*_*PGK1*_ and *P*_*TDH3*_ promoters respectively), were tested for LP production by expressing *T79LPS*^*M593I/Y700F*^, resulting in the formation of the W10 and W11 strains, respectively. LP production in W10 and W11 was 28.6 and 83.9 μg/L, respectively. GGPP was produced via FPP and IPP condensation, and *IDI1* over-expression did not benefit IPP accumulation. Therefore, WTE was applied for subsequent LA production processes. Although native *BTS1* was further over-expressed, it could not compete with ERG9 for FPP [14]. ERG9 is a crucial enzyme which initiates the sterol synthesis and is often down-regulated by replacing its native promoter with *P*_*MET3*_ [26, 27]. In our study, *ERG9* down-regulation in the W12 strain increased LP production to 799.2 μg/L, which was 8.5-fold higher than that in its parental strain W11. In order to continuously improve precursor LP production, the *BTS1* and the *T79LPS*^*M593I/Y700F*^ genes were co-expressed in the W9 *rDNA* site, thereby resulting in the strain WM, which could produce 49.21 mg/L of LP (Fig. 4b).

To produce LA in the WM strain, *CYP720B1* and *CPRs* were co-expressed. Three CPRs from different sources (*AtCPR1* from *Arabidopsis thaliana*, *AtCPR2* from *A. thaliana*, and *TcCPR* from *Taxus cuspidate*) were selected for co-expression with *CYP720B1* in WM, resulting in the WM1, WM2, and WM3 strains, respectively. As shown in Fig. 4a and c, strain WM1 could produce 17.49 mg/L of LA and the highest LA production (23.13 mg/L) was achieved by WM3. However, *CYP720B1*-*AtCRP2* co-expression (WM2) failed to convert LP to LA. Compared to AtCPR1, AtCPR2 contains a poly-serine N-terminal sequence [28] that may lead to the unsuccessful pairing with CYP720B1. Generally, plant P450 s depend on cytochrome P450 reductase (CPR) and NADPH to initiate electron transfer. CPR is essential and important to its activity. An adaptation CPR often accompanies heterologously-expressed cytochrome P450 s in *S. cerevisiae* due to the lack of compatible redox partners [29]. The CPR and plant P450 s interaction efficiency is somewhat modulated depending on the CPR homolog present [28]. CPR is even reported to influence the metabolite pattern of P450 [30] However, it was hard to determine the native CPRs for specific P450 s and thus, it became necessary to try different available CPRs. In a recent study, glycyrrhetinic acid, a triterpenoid compound found in licorice, was efficiently synthesized by pairing CPRs from various plant sources in *S. cerevisiae.* A CPR from *Glycyrrhiza uralensis* was identified and applied to transfer electrons to the glycyrrhetinic acid synthesis pathway, thereby achieving highest glycyrrhetinic acid titer [31].

In order to improve CYP720B1 expression, *CYP720B1* and *TcCPR* genes were integrated into the WM multi-copy δ site, resulting in the WMM strain. Consequently, LA production in WMM increased to 45.24 mg/L.

### Production of levopimaric acid in fed-batch fermentation

**Fig. 5:**
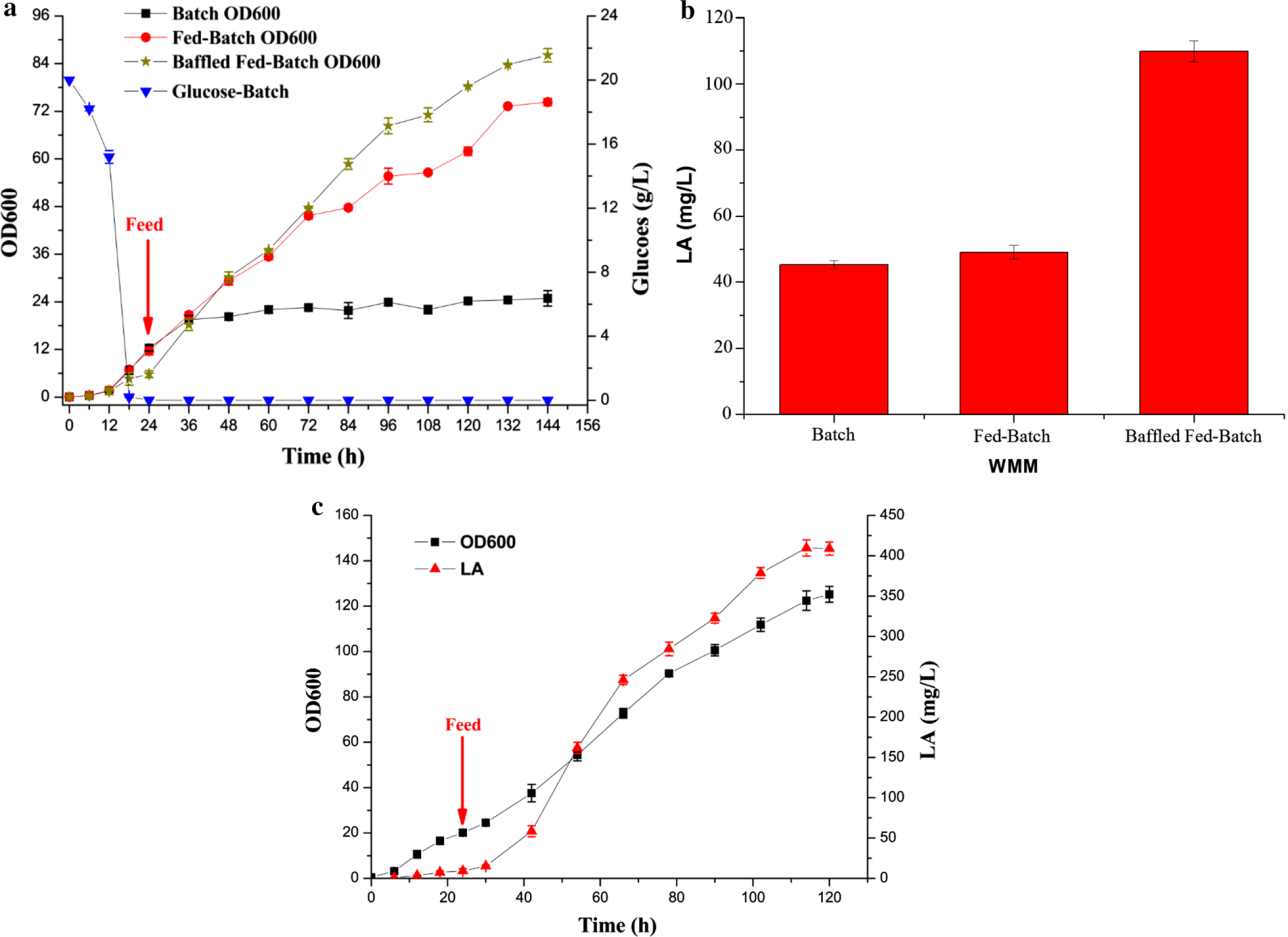
Production of LA via fed-batch fermentation. **a** Fed-batch fermentation of WMM in shake-flasks. **b** LA production of WMM in shake-flask fermentation. **c** Fed-batch fermentation of WMM in a 5-L bioreactor. Error bars represent the standard deviation from three independent experiments

Strain WMM was used for fed-batch fermentation. As shown in Fig. 5a, the OD_600_ of WMM increased to 74.3, which was about a threefold increase when compared to batch fermentation. The production of LA (49.21 mg/L) also slightly increased compared to the batch fermentation (Fig. 5b). However, we found that the content of the unknown peak (Fig. 4a Peak 3) increased, and we speculate that it could be an intermediate product since LP forms LA via a three-step oxidation reaction. Accumulation of the unknown intermediate product indicated that dissolved oxygen (DO) might be the rate-limiting step for LP production. To test this, baffled-bottom flasks were applied for fed-batch cultivation, which significantly increased LA production by 2.4-fold to 109.83 mg/L. According to WMM shake-flask fed-batch fermentation results, DO was maintained above 35% when it was applied to the scale-up process in the 5-L bioreactor and feed solution was added after 24 h. Consequently, the production of LA was increased to 400.31 mg/L (Fig. 5c). WMM strain performance in producing LA from glucose is calculated in Table 1, which shows the yield and productivity in the 5 L fermenter was higher than all the flasks. This proves that DO control is necessary for efficiently producing LA.

**Table 1:** The titer, yield, and productivity of strain WMM

Fermentation method	Titer (mg/L)	Yield (mg/g glucose)	Productivity (mg/L/h)
Batch in shake-flask	45.24 ± 1.47	2.26 ± 0.07	0.31 ± 0.01
Fed-batch in shake-flask	49.21 ± 2.13	0.46 ± 0.02	0.34 ± 0.02
Fed-batch in baffled-bottom flask	109.83 ± 3.25	1.04 ± 0.04	0.76 ± 0.02
Fed-batch in 5 L fermenter	400.31 ± 8.11	3.77 ± 0.14	3.33 ± 0.07

## Conclusions

Here we report that *S. cerevisiae* could be engineered to de novo produce LP and LA from glucose by expressing LPS, CYP720B1, and TcCPR. It is necessary to combine metabolic engineering and protein engineering strategies to solve potential metabolic bottlenecks in microbial cell factories when they are used for natural products production. The strategies reported here can also be applied for synthesizing other valuable diterpenoids in yeast, especially for the synthesis pathway containing cytochrome P450 s.

## Methods

### Strains and media

*Saccharomyces cerevisiae* W303-1a was selected as the original strain to construct and optimize LA production. *LPS* gene (GenPept: Q947C4.1) was synthesized by GenScript (Nanjing, China) with codon optimization for *S.cerevisiae*, and the *CYP720B1* (GenPept: Q50EK6.1), *AtCPR1* (GenPept: NP194183.1), *AtCPR2* (GenPept: CAA46815.1), and *TcCPR* (GenPept: AAT76449.1) genes kindly provided by Professor Yingjin Yuan (School of Chemical Engineering and Technology, Tianjin University, China) were also codon optimized for *S.cerevisiae*. Promoters, terminators, selectable markers and other genes were all amplified from the *S.cerevisiae* genomic DNA. Synthetic complete (SC) medium, lacking appropriate amino acids, was used to screen yeast transformants and yeast extract peptone dextrose medium (YPD; 2% peptone, 2% glucose, and 1% yeast extract) was used to cultivate yeast.

### Yeast expression cassettes construction and transformation

Expression cassettes containing the promoter, gene and terminator were constructed by fusion PCR. Site-directed mutation and N-terminal truncation of LPS were performed via overlap-extension PCR. *S. cerevisiae* transformations were performed by the LiAc method, and the DNA fragments were inserted into the yeast genome via the homologous recombination method [32]. Strains constructed in this study are listed in Table 2, whereas the expression cassettes and primers used for strain construction are separately shown in Additional file 1: Fig. S1, Tables S1 and S2.

**Table 2:** Strains involved in this study

Strains	Description	Source
W303-1a	*MATa; leu2*-*3112; trp1*-*1; can1*-*100; ura3*-*1; ade2*-*1; his3*-*11,15*	Our lab
WTI	W303-1a*; δ*:: *P*_*PGK1*_- *tHMG1*-*T*_*PGK1*_*, T*_*TDH3*_-*IDI1*-*T*_*ADH1*_	This study
WTI-BE	W303-1a*; δ*:: *P*_*PGK1*_- *tHMG1*-*T*_*PGK1*_*, T*_*TDH3*_-*IDI1*-*T*_*ADH1*_*, P*_*PGK1*_-*BTS1*-*GGGS*-*ERG20*- *T*_*CYC1*_	This study
W1	W303-1a; *δ*:: *P*_*PGK1*_- *tHMG1*-*T*_*PGK1*_*, T*_*TDH3*_-*IDI1*-*T*_*ADH1*_*, P*_*PGK1*_-*BTS1*-*GGGS*-*ERG20*- *T*_*CYC1*_; *ura3:: P*_*TEF1*_-*LPS*-*T*_*ADH2*_	This study
W2	W303-1a; *δ*:: *P*_*PGK1*_- *tHMG1*-*T*_*PGK1*_*, T*_*TDH3*_-*IDI1*-*T*_*ADH1*_*, P*_*PGK1*_-*BTS1*-*GGGS*-*ERG20*- *T*_*CYC1*_; *ura3:: P*_*TEF1*_-*LPS*^*MM*^-*T*_*ADH2*_	This study
W3	W303-1a; *δ*:: *P*_*PGK1*_- *tHMG1*-*T*_*PGK1*_*, T*_*TDH3*_-*IDI1*-*T*_*ADH1*_*, P*_*PGK1*_-*BTS1*-*GGGS*-*ERG20*- *T*_*CYC1*_; *ura3:: P*_*TEF1*_-*T40LPS*-*T*_*ADH2*_	This study
W4	W303-1a; *δ*:: *P*_*PGK1*_- *tHMG1*-*T*_*PGK1*_*, T*_*TDH3*_-*IDI1*-*T*_*ADH1*_*, P*_*PGK1*_-*BTS1*-*GGGS*-*ERG20*- *T*_*CYC1*_; *ura3:: P*_*TEF1*_-*T40LPS*^*MM*^-*T*_*ADH2*_	This study
W5	W303-1a; *δ*:: *P*_*PGK1*_- *tHMG1*-*T*_*PGK1*_*, T*_*TDH3*_-*IDI1*-*T*_*ADH1*_*, P*_*PGK1*_-*BTS1*-*GGGS*-*ERG20*- *T*_*CYC1*_; *ura3:: P*_*TEF1*_-*T60LPS*-*T*_*ADH2*_	This study
W6	W303-1a; *δ*:: *P*_*PGK1*_- *tHMG1*-*T*_*PGK1*_*, T*_*TDH3*_-*IDI1*-*T*_*ADH1*_*, P*_*PGK1*_-*BTS1*-*GGGS*-*ERG20*- *T*_*CYC1*_; *ura3:: P*_*TEF1*_-*T60LPS*^*MM*^-*T*_*ADH2*_	This study
W7	W303-1a; *δ*:: *P*_*PGK1*_- *tHMG1*-*T*_*PGK1*_*, T*_*TDH3*_-*IDI1*-*T*_*ADH1*_*, P*_*PGK1*_-*BTS1*-*GGGS*-*ERG20*- *T*_*CYC1*_; *ura3:: P*_*TEF1*_-*T79LPS*-*T*_*ADH2*_	This study
W8	W303-1a; *δ*:: *P*_*PGK1*_- *tHMG1*-*T*_*PGK1*_*, T*_*TDH3*_-*IDI1*-*T*_*ADH1*_*, P*_*PGK1*_-*BTS1*-*GGGS*-*ERG20*- *T*_*CYC1*_; *ura3:: P*_*TEF1*_-*T79LPS*^*MM*^-*T*_*ADH2*_	This study
WTE	W303-1a*; ade2*:: *P*_*PGK1*_- *tHMG1*-*T*_*PGK1*_*, T*_*TDH3*_-*ERG20*-*T*_*ERG20*_	This study
W9	W303-1a*; ade2*:: *P*_*PGK1*_- *tHMG1*-*T*_*PGK1*_*, T*_*TDH3*_-*ERG20*-*T*_*ERG20*_*; P*_*ERG9*_*::P*_*MET3*_-*ERG9*	This study
W10	W303-1a; *δ*:: *P*_*PGK1*_- *tHMG1*-*T*_*PGK1*_*, T*_*TDH3*_-*IDI1*-*T*_*ADH1*_*, ura3:: P*_*TEF1*_-*T79LPS*^*MM*^-*T*_*ADH1*_*, P*_*TEF1*_-*BTS1*-*T*_*ADH2*_	This study
W11	W303-1a; *ade2*:: *P*_*PGK1*_- *tHMG1*-*T*_*PGK1*_*, T*_*TDH3*_-*ERG20*-*T*_*ERG20*_*, ura3:: P*_*TEF1*_-*T79LPS*^*MM*^-*T*_*ADH1*_*, P*_*TEF1*_-*BTS1*-*T*_*ADH2*_	This study
W12	W303-1a*; ade2*:: *P*_*PGK1*_- *tHMG1*-*T*_*PGK1*_*, T*_*TDH3*_-*ERG20*-*T*_*ERG20*_*; P*_*ERG9*_*::P*_*MET3*_-*ERG9; ura3:: P*_*TEF1*_-*T79LPS*^*MM*^-*T*_*ADH1*_*, P*_*TEF1*_-*BTS1*-*T*_*ADH2*_	This study
WM	W303-1a*; ade2*:: *P*_*PGK1*_- *tHMG1*-*T*_*PGK1*_*, T*_*TDH3*_-*ERG20*-*T*_*ERG20*_*; P*_*ERG9*_*::P*_*MET3*_-*ERG9; rDNA:: P*_*TEF1*_-*T79LPS*^*MM*^-*T*_*ADH1*_*, P*_*TEF1*_-*BTS1*-*T*_*ADH2*_	This study
WM1	W303-1a*; ade2*:: *P*_*PGK1*_- *tHMG1*-*T*_*PGK1*_*, T*_*TDH3*_-*ERG20*-*T*_*ERG20*_*; P*_*ERG9*_*::P*_*MET3*_-*ERG9; rDNA:: P*_*TEF1*_-*T79LPS*^*MM*^-*T*_*ADH1*_*, P*_*TEF1*_-*BTS1*-*T*_*ADH2*_*; his3:: P*_*PGK1*_-*CYP720B1*-*T*_*ADH1*_, *P*_*TDH3*_-*AtCPR1*-*T*_*TDH2*_	This study
WM2	W303-1a*; ade2*:: *P*_*PGK1*_- *tHMG1*-*T*_*PGK1*_*, T*_*TDH3*_-*ERG20*-*T*_*ERG20*_*; P*_*ERG9*_*::P*_*MET3*_-*ERG9; rDNA:: P*_*TEF1*_-*T79LPS*^*MM*^-*T*_*ADH1*_*, P*_*TEF1*_-*BTS1*-*T*_*ADH2*_*; his3:: P*_*PGK1*_-*CYP720B1*-*T*_*ADH1*_, *P*_*TDH3*_-*AtCPR2*-*T*_*TDH2*_	This study
WM3	W303-1a*; ade2*:: *P*_*PGK1*_- *tHMG1*-*T*_*PGK1*_*, T*_*TDH3*_-*ERG20*-*T*_*ERG20*_*; P*_*ERG9*_*::P*_*MET3*_-*ERG9; rDNA:: P*_*TEF1*_-*T79LPS*^*MM*^-*T*_*ADH1*_*, P*_*TEF1*_-*BTS1*-*T*_*ADH2*_*; his3:: P*_*PGK1*_-*CYP720B1*-*T*_*ADH1*_, *P*_*TDH3*_-*TcCPR*-*T*_*TDH2*_	This study
WMM	W303-1a*; ade2*:: *P*_*PGK1*_- *tHMG1*-*T*_*PGK1*_*, T*_*TDH3*_-*ERG20*-*T*_*ERG20*_*; P*_*ERG9*_*::P*_*MET3*_-*ERG9; rDNA:: P*_*TEF1*_-*T79LPS*^*MM*^-*T*_*ADH1*_*, P*_*TEF1*_-*BTS1*-*T*_*ADH2*_*; δ:: P*_*PGK1*_-*CYP720B1*-*T*_*ADH1*_, *P*_*TDH3*_-*TcCPR*-*T*_*TDH2*_	This study

### Yeast strain cultivation and fermentation

A single clone was picked from the SC plate, inoculated into 5 mL of YPD medium, and cultivated overnight at 30 °C and 220 rpm. The overnight culture was then inoculated into Erlenmeyer flasks containing 30 mL of YPD medium at an initial OD_600_ of 0.05 and cultured at 30 °C and 220 rpm for 5 days. For LP production, 3 mL of n-dodecane was added after inoculation. Met (0.3 g/L) was added to the medium when the native promoter of *Erg9* was replaced with *P*_*MET3*_ after 24 h. All Erlenmeyer flask experiments were performed in triplicate. The WMM strain was applied for batch and fed-batch fermentation process. A single colony obtained from the plate was inoculated into 30 mL of YPD medium and cultivated overnight at 220 rpm and 30 °C. For shake-flask fed-batch fermentation, the overnight culture was transferred into a 500-mL flask containing 60 mL of YPD medium with an initial OD600 of 0.05. After 24 h, 1.5 mL of glucose solution (800 g/L of glucose), 1 mL of glutamate solution (300 g/L of glutamate sodium) and 2 mL of mixed feed solution were added every 12 h. Feeding was stopped at 96 h. The mixed feed solution (1 L) contained KH_2_PO_4_ (9 g), K_2_SO_4_ (3.5 g), Na_2_SO_4_ (0.28 g), MgSO_4_∙7H_2_O (5.12 g), 10 mL of microelement stock solution (containing ZnSO_4_∙7H_2_O (10.2 g/L), EDTANa_2_∙2H_2_O (15 g/L), FeSO_4_∙7H_2_O (5.12 g/L), CuSO_4_ (0.5 g/L), MnCl_2_∙4H_2_O (0.5 g/L), CoCl_2_∙6H_2_O (0.86 g/L), CaCl_2_∙2H_2_O (3.84 g/L), and Na_2_MoO_4_∙2H_2_O (0.56 g/L)), and 12 mL of vitamin stock solution (containing inositol (25 g/L), biotin (0.05 g/L), niacin (1 g/L), calcium pantothenate (1 g/L), thiamine HCl (1 g/L), pyridoxol HCl (1 g/L), and p-aminobenzoic acid (0.2 g/L)).

For the 5-L scale-up fermentation process, the overnight yeast culture was inoculated into new 500 mL Erlenmeyer flasks containing 100 mL of YPD medium with an initial OD_600_ of 0.05 and cultured at 30 °C, and 220 rpm for 24 h. The culture was then used to seed a 5-L bioreactor (Shanghai BaiLun Bio-Technology) containing 2 L of YPD medium. Fermentation was carried out at 30 °C with an air flow rate of 2 L/min. The pH was automatically controlled at 5.5 with 2 M NaOH and 5 N H_2_SO_4_. DO, in correlation with stirring, was maintained above 35%. The feed solution, which was fed every 12 h to a glucose concentration of 20 g/L after 24 h, was the same as the shake-flask fed-batch fermentation process.

### Metabolite extraction

Extracellular LP was extracted using n-dodecane and intracellular LP was extracted using n-hexane, while the cells were centrifugally collected and broken. Intracellular and extracellular LA was extracted using ethyl acetate. Glucose was measured by bio-analyzer (SBA-40C, Shandong Academy of Sciences, China) following the manufacturer’s instructions. OD600 was measured via a spectrophotometer (Oppler, 752 N, China).

### Homology modeling of LPS

Homology modeling of LPS was performed by the I-TASSER server (http://zhanglab.ccmb.med.umich.edu/I-TASSER/). The template used was abietadiene synthase from *A. grandis* (PDB: 3s9v) as it had the highest homology (67%) with LPS. The amino acid sequences of LPS and the template were submitted to I-TASSER to obtain the predicted model.

### Identification and quantification of LP and LA

LP was identified and quantified by GC or GC–MS. Dodecane extracts (1 μL) separated from the yeast bi-phase culture, were filtrated and analyzed by GC–MS using an Agilent Technologies 7890A GC system equipped with a 5975C insert 143 XL EI/CI MSD Detector with an HP-5 ms chromatographic column. Helium was used as the carrier gas at a flow rate of 1 mL/min. The oven temperature was kept at 150 °C for 5 min, increased to 250 °C at the rate of 5 °C/min, and finally held at 250 °C for 5 min. The injector and detector temperatures were 250 and 260 °C, respectively. LP amounts were determined using the internal standard 1-eicosene [33–35].

Ethyl acetate extracts were analyzed by LC–MS or LC in order to identify and quantify LA. LC–MS was performed on a Thermo Fisher LCQ Advantage MAX instrument equipped with an electrospray ionization detector with a C18 column (4.6 mm × 250 mm). Samples were injected at a column flow of 1 mL/min in the mobile phase (methanol: water: formic acid = 87:13:0.02). LA amounts were determined using standard curves.

## Additional file

**Additional file 1: Table S1.** Primers used in this study. **Table S2.** Primers used for cassettes construction in this study. **Figure S1.** Construction of gene expression cassettes and yeast strains.
