# Supplementary material for: Heterologous production of levopimaric acid in Saccharomyces cerevisiae
Source: Microb Cell Fact. 2018 Jul 18;17:114. doi: 10.1186/s12934-018-0964-1 (PMC6050663; doi:10.1186/s12934-018-0964-1)
Supplement: Supplementary file 1 — Additional file 1: Table S1. Primers used in this study. Table S2. Primers used for cassettes construction in this study. Figure S1. Construction of gene expression cassettes and yeast strains. [file 12934_2018_964_MOESM1_ESM.docx]

**Additional file for**

**Heterologous production of** **levopimaric acid in *Saccharomyces cerevisiae***

**Ting Liu^a#^, Chuanbo Zhang^a#^, Wenyu Lu^a,b,c *^**

^a^ School of Chemical Engineering and Technology, Tianjin University, Tianjin 300072, PR China;

^b^ Key Laboratory of System Bioengineering (Tianjin University), Ministry of Education, Tianjin, 300072, PR China;

^c^ Collaborative Innovation Center of Chemical Science and Engineering (Tianjin), SynBio Res Platform, Tianjin, 300072, PR China.

^#^ These authors contributed equally to this work.

* Correspondence to School of Chemical Engineering and Technology, Tianjin University, Tianjin 300072, PR China. Tel: +86-22-27892132, Fax: +86-22-27400973.

E-mail address: wenyulu@tju.edu.cn (W. Lu)

**Table S1** Primers used in this study

| **Serial No.** | **Name** | | **Primer sequence (5-3)** |
| --- | --- | --- | --- |
| ***tHMGI* and *IDI1* gene expression cassettes construction** | | | |
| 1 | δ2-F | | GCTTCGGTTACTTCTAAGGAAGTCCACACAAATCAAGATCCGTTAG |
| 2 | PGK1-δ2-R | | GTTATAATATCTGTGCGTCTTGAGTTGAAGTCAGGAATCTAAAATATTGGAAAGTCATTAGGTGAGGTT |
| 3 | δ2-PGK1-F | | TATGTCAGACCACCACCAATGTTAACCTCACCTAATGACTTTCCAATATTTTAGATTCCTGACTTCAAC |
| 4 | tHMG1-PGK1-R | | ATTGGTTAAAACTGGCATTGTTTTATATTTGTTGTAAAAAGTAG |
| 5 | PGK1-tHMG1-F | | CAACAAATATAAAACAATGCCAGTTTTAACCAATAAAACAGTC |
| 6 | PGK1T-tHMG1-R | | CTATCGATTTCAATTCAATTCAATTTAGGATTTAATGCAGGTGAC |
| 7 | tHMG1- PGK1T-F | | GTCACCTGCATTAAATCCTAAATTGAATTGAATTGAAATCGATAG |
| 8 | TDH3-PGK1T-R | | TAACATTCAACGCTAGTATCACTATACTGGATCTAAAGAGTACAATAG |
| 9 | PGKT-TDH3-F | | CTCTTTAGATCCAGTATAGTGATACTAGCGTTGAATGTTAGCG |
| 10 | IDI-TDH3-R | | CTATTGTTGTCGGCAGTCATTTTGTTTGTTTATGTGTG |
| 11 | TDH3-IDI-F | | CACACATAAACAAACAAAATGACTGCCGACAACAATAG |
| 12 | ADHI-IDI-R | | ATCATAAATCATAAGAAATTCGCTTATAGCATTCTATGAATTTGCC |
| 13 | IDI-ADH1-F | | GGCAAATTCATAGAATGCTATAAGCGAATTTCTTATGATTTATGAT |
| 14 | LEU2-ADH1-R | | CATGAACAAGGAAGTACAGGACAGATCATGATACATAAAAGCGATATAAC |
| 15 | ADH1-LEU2-F | | GTTATATCGCTTTTATGTATCATGATCTGTCCTGTACTTCCTTGTTCATGTG |
| 16 | δ1-LEU2-R | | GATAGTTGATTTCTATTCCAACAGGAAATGGTTCAAGAAGGTATTGAC |
| 17 | LEU2-δ1-F | | GTCAATACCTTCTTGAACCATTTCCTGTTGGAATAGAAATCAACTATC |
| 18 | δ1-R | | CACAGGCGCTACCATGAGAATTG |
| ***Bts1-Erg20* fusion protein expression cassettes construction** | | | |
| 19 | δ2-F | | GCTTCGGTTACTTCTAAGGAAGTCCACACAAATCAAGATCCGTTAGAG |
| 20 | Pgk1p-δ2-R | | GTTGAAGTCAGGAATCTAAAATATTGGAAAGTCATTAGGTGAGG |
| 21 | δ2-Pgk1p-F | | CCTCACCTAATGACTTTCCAATATTTTAGATTCCTGACTTCAAC |
| 22 | Bts1-Pgk1p-R | | GCTCATCTATCTTGGCCTCCATTGTTTTATATTTGTTGTAAAAAG |
| 23 | Pgk1p-Bts1-F | | CTTTTTACAACAAATATAAAACAATGGAGGCCAAGATAGATGAGC |
| 24 | Erg20-Bts1-R | | CTAATTTCTTTTTCTGAAGCCATAGAACCACCACCCAATTCGGATAAGTGGTCTATTA |
| 25 | Bts1-Erg20-F | | TAATAGACCACTTATCCGAATTGGGTGGTGGTTCTATGGCTTCAGAAAAAGAAATTAG |
| 26 | Cyc1t -Erg20-R | | GATGCGGCCCTCTAGGATCAGCGGCTATTTGCTTCTCTTGTAAAC |
| 27 | Erg20-Cyc1t-F | | GTTTACAAGAGAAGCAAATAGCCGCTGATCCTAGAGGGCCGCATC |
| 28 | Trp1-Cyc1t-R | | CTGTCCCTAACTCGGTCTCTTTCGCGCGTTGGCCGATTCATTAATG |
| 29 | Cyc1t-Trp1-F | | CATTAATGAATCGGCCAACGCGCGAAAGAGACCGAGTTAGGGACAG |
| 30 | δ1-trp1-R | | ATAGTTGATTTCTATTCCAACAGTCAGCGTAGAAGATAATAGT |
| 31 | trp1-δ1-F | | ACTATTATCTTCTACGCTGACTGTTGGAATAGAAATCAACTAT |
| 32 | δ1-R | | CACAGGCGCTACCATGAGAATTGGGTGAATGTTGAGATAATTGTTG |
|  |  | | ***LPS*(M593I-Y700F) cassettes construction** |
| 33 | Tef1p-LPS-F | | ATCTAAGTTTTAATTACAAAATGGCAGGTGTTTTGTTCGC |
| 34 | M593I-LPS-R | | ACTGCAACTGAAAAGTAGATTTCAACTGGTCTTTGTC |
| 35 | LPS-M593I-F | | GACAAAGACCAGTTGAAATCTACTTTTCAGTTGCAGT |
| 36 | Adh2t-LPS-R | | GTAAAGACATAAGAGATCCGCTTATGCAACTGGATCGAACAA |
| 37 | LPS-Y700F-F | | GAAGGTTTGTTAGCATCATTCACTAAAGAAGCTGAATGG |
| 38 | Y700F-LPS-R | | CCATTCAGCTTCTTTAGTGAATGATGCTAACAAACCTTC |
| ***LPS*，*LPS*(M593I-Y700F)，*T40LPS*，*T40******LPS*(M593I-Y700F)，*T60LPS*，*T60LPS*(M593I-Y700F)，*T79LPS* and *T79LPS*(M593I-Y700F) expression cassettes construction** | | | |
| 39 | Ura3B-F | | AATGCACACGGTGTGGTGGGCCCAGG |
| 40 | Tef1p -Ura3B-R | | TTGAAGCTATGGTGTGTGGGGGATCACTGGGTAATAACTGATATAATTAAATTGAAGCTC |
| 41 | Ura3B-Tef1p-F | | GAGCTTCAATTTAATTATATCAGTTATTACCCAGTGATCCCCCACACACCATAGCTTCAA |
| 42 | LPS-Tef1p-R | | GCGAACAAAACACCTGCCATTTTGTAATTAAAACTTAGAT |
| 43 | Tef1p-LPS-F | | ATCTAAGTTTTAATTACAAAATGGCAGGTGTTTTGTTCGC |
| 44 | T40LPS -Tef1p-R | | TGATCTAACACAATGTTGTGCCATTTTGTAATTAAAACTTAGATTA |
| 45 | Tef1p-T40 LPS-F | | TAATCTAAGTTTTAATTACAAAATGGCACAACATTGTGTTAGATCA |
| 46 | T60LPS -Tef1p-R | | CTGGTCTTGTTTCTGCAGCAGACATTTTGTAATTAAAACTTAGATT |
| 47 | Tef1p-T60 LPS-F | | TAATCTAAGTTTTAATTACAAAATGTCTGCTGCAGAAACAAGACCAG |
| 48 | T79LPS -Tef1p-R | | CTGGATGATAATCTGCATTCAACATTTTGTAATTAAAACTTAGATTA |
| 49 | Tef1p-T79LPS-F | | TAATCTAAGTTTTAATTACAAAATGTTGAATGCAGATTATCATCCAG |
| 50 | Adh2-LPS-R | | GTAAAGACATAAGAGATCCGCTTATGCAACTGGATCGAACAA |
| 51 | LPS-Adh2t-F | | TTGTTCGATCCAGTTGCATAAGCGGATCTCTTATGTCTTTAC |
| 52 | Ura3A-Adh2t-R | | ATTGAAAAGCTTTATGGACCCTGATGAAATTATAGGGTGGACGTC |
| 53 | Adh2t- Ura3A-F | | GACGTCCACCCTATAATTTCATCAGGGTCCATAAAGCTTTTCAAT |
| 54 | Ura3A-R | | CGTAATGTCTGCCCATTCTGCTATTCTGTATACACCCGC |
| ***tHMGI* and *ERG20* gene expression cassettes construction** | | | |
| 55 | ADE2L-F | TAACGCCGTATCGTGATTAACGTATTAC | |
| 56 | PGK1-ADE2L-R | GAAGTCAGGAATCTAAAATACATGAAATGCTCCATAATATTG | |
| 57 | Ade2L-PGK1-F | ATATTATGGAGCATTTCATGTATTTTAGATTCCTGACTTC | |
| 58 | tHMG1-PGK1-R | ATTGGTTAAAACTGGCATTGTTTTATATTTGTTGTAAAAAGTAG | |
| 59 | PGK1-tHMG1-F | CAACAAATATAAAACAATGCCAGTTTTAACCAATAAAACAGTC | |
| 60 | PGK1T-tHMG1-R | CTATCGATTTCAATTCAATTCAATTTAGGATTTAATGCAGGTGAC | |
| 61 | tHMG1-PGK1T-F | GTCACCTGCATTAAATCCTAAATTGAATTGAATTGAAATCGATAG | |
| 62 | TDH3-PGK1T-R | TAACATTCAACGCTAGTATCACTATACTGGATCTAAAGAGTACAATAG | |
| 63 | PGKT-TDH3-F | CTCTTTAGATCCAGTATAGTGATACTAGCGTTGAATGTTAGCG | |
| 64 | ERG20-TDH3-R | ATTTCTTTTTCTGAAGCCATTTTGTTTGTTTATGTGTGTTTATTC | |
| 65 | TDH3-ERG20-F | ACACACATAAACAAACAAAATGGCTTCAGAAAAAGAAATTAG | |
| 66 | LEU2-ERG20-R | GGCATGCAAGCTTGCAATGTTCGAGAGATGAGGTC | |
| 67 | ERG20-LEU2-F | TCTCGAACATTGCAAGCTTGCATGCCTGC | |
| 68 | ADE2R-LEU2-R | CTAGACAAGAACGATGAATTCGAGCTC | |
| 69 | LEU2-ADE2R-F | CTCGAATTCATCGTTCTTGTCTAGATGGAGTAG | |
| 70 | ADE2R-R | CGCTATCCTCGGTTCTGCATTG | |
|  |  | **The native *ERG9* promoter of WTE was replaced by Pmet3** | |
| 71 | 9A-F | AGTGCAGCTCAGAGCCCCCAGC | |
| 72 | ade2-9A-R | CACGATACGGCGTTATGTGTGTGTGTGATATGTG | |
| 73 | A-ADE2-F | CATATCACACACACACATAACGCCGTATCGTGATTAACG | |
| 74 | Met3-ADE2-R | CTTATACCACTACGTACATATCGTTGCGCTATCCTCGGTTCTGCATTG | |
| 75 | ade2-met3-F | CAATGCAGAACCGAGGATAGCGCAACGATATGTACGTAGTGGTATAAG | |
| 76 | 9B-Met3-R | GCCAATTGTAATAGCTTTCCCATGTTAATTATACTTTATTCTTG | |
| 77 | Met3-9B-F | CAAGAATAAAGTATAATTAACATGGGAAAGCTATTACAATTGGC | |
| 78 | 9B-R | ACACGTCGTAGTCGTGGACGGTTTGCAAC | |
| T79LPS(M593I-Y700F) and Bts1 gene expression cassettes were integrated into the *ura3* site | | | |
| 79 | Ura3B-F | | AATGCACACGGTGTGGTGGGCCCAGG |
| 80 | Tef1p-Ura3B-R | | TTGAAGCTATGGTGTGTGGGGGATCACTGGGTAATAACTGATATAATTAAATTGAAGCTC |
| 81 | Ura3B-Tef1p-F | | GAGCTTCAATTTAATTATATCAGTTATTACCCAGTGATCCCCCACACACCATAGCTTCAA |
| 82 | T79LPS-Tef1p-R | | CTGGATGATAATCTGCATTCAACATTTTGTAATTAAAACTTAGATTA |
| 83 | Tef1p-T79LPS-F | | TAATCTAAGTTTTAATTACAAAATGTTGAATGCAGATTATCATCCAG |
| 84 | Adh1t-LPS-R | | AAATCATAAATCATAAGAAATTCGCTTATGCAACTGGATCGAACAA |
| 85 | LPS-Adh1t-F | | TTGTTCGATCCAGTTGCATAAGCGAATTTCTTATGATTTATGATTT |
| 86 | Tef1p-Adh1t-R | | GCTATGGTGTGTGGGGGATCACTAGATCATGATACATAAAAGCG |
| 87 | Adh1t-Tef1p-F | | CGCTTTTATGTATCATGATCTAGTGATCCCCCACACACCATAGC |
| 88 | Bts1-Tef1p-R | | CTCATCTATCTTGGCCTCCATTTTGTAATTAAAACTTAGATTAG |
| 89 | Tef1p-Bts1-F | | CTAATCTAAGTTTTAATTACAAAATGGAGGCCAAGATAGATGAG |
| 90 | Adh2t-Bts1-R | | ATCGTAAAGACATAAGAGATCCGCTCACAATTCGGATAAGTGGTCTA |
| 91 | Bts1-Adh2t-F | | TAGACCACTTATCCGAATTGTGAGCGGATCTCTTATGTCTTTACGAT |
| 92 | Ura3A-Adh2t-R | | ATTGAAAAGCTTTATGGACCCTGATGAAATTATAGGGTGGACGTC |
| 93 | Adh2t-Ura3A-F | | GACGTCCACCCTATAATTTCATCAGGGTCCATAAAGCTTTTCAAT |
| 94 | Ura3A-R | | CGTAATGTCTGCCCATTCTGCTATTCTGTATACACCCGC |
| ***T79LPS(M593I-Y700F)* and *Bts1* gene expression cassettes were integrated into *rDNA* site** | | | |
| 95 | R1-F | | CCGGGGCACCTGTCACTTTGGAAAAAAAATATACGCTAAGA |
| 96 | Tef1p-R1-R | | CTATGGTGTGTGGGGGATCACTTTTCCTCTAATCAGGTTCCACC |
| 97 | R1-Tef1p-F | | GGTGGAACCTGATTAGAGGAAAAGTGATCCCCCACACACCATAG |
| 98 | R2-Ura3-R | | GACTTACGTTTGCTACTCTCATGGGTAATAACTGATATAATTAA |
| 99 | Ura3-R2-F | | TTAATTATATCAGTTATTACCCATGAGAGTAGCAAACGTAAGTC |
| 100 | R2-R | | GCGGAAAATACGGAAACGCGCGGGAACATACAA |
| ***CYP720B1*, *AtCPR1* , *AtCPR2*, *TcCPR* gene expression cassettes construction** | | | |
| 101 | His3B-F | | CATCACACCACTGAAGACTGCGGG |
| 102 | Pgk1p-His3B-R | | GAGTTGAAGTCAGGAATCTAAAATATCGAGTTCAAGAGAAAAAAAAAG |
| 103 | His3B-pgk1p-F | | CTTTTTTTTTCTCTTGAACTCGATATTTTAGATTCCTGACTTCAACTC |
| 104 | CYP720B1-Pgk1p-R | | ACAAAGAAATTTGATCAGCCATTGTTTTATATTTGTTGTAAAAAG |
| 105 | Pgk1p-CYP720B1-F | | CTTTTTACAACAAATATAAAACAATGGCTGATCAAATTTCTTTGT |
| 106 | Adh1t-CYP720B1-R | | ATCATAAATCATAAGAAATTCGCTTACTCTCTATTGTACAACCTC |
| 107 | CYP720B1-Adh1t-F | | GAGGTTGTACAATAGAGAGTAAGCGAATTTCTTATGATTTATGAT |
| 108 | Tdh3p-Adh1t-R | | GCTAACATTCAACGCTAGTATAGATCATGATACATAAAAGCG |
| 109 | Adh1t-Tdh3p-F | | TCGCTTTTATGTATCATGATCTATACTAGCGTTGAATGTTAGC |
| 110 | AtCPR1-Tdh3p-R | | CTGTATTGGAATTGGCTTGCATTTTGTTTGTTTATGTGTGTTTA |
| 111 | Tdh3p-AtCPR1-F | | TAAACACACATAAACAAACAAAATGACTTCTGCATTATACGC |
| 112 | AtCPR2-Tdh3p-R | | GATGATGATGAAGATGATGACATTTTGTTTGTTTATGTGTGTTTA |
| 113 | Tdh3p-AtCPR2-F | | TAAACACACATAAACAAACAAAATGTCATCATCTTCATCATCATC |
| 114 | TcCPR-Tdh3p-R | | CTGTATTGGAATTGGCTTGCATTTTGTTTGTTTATGTGTGTTTA |
| 115 | Tdh3p-TcCPR-F | | TAAACACACATAAACAAACAAAATGCAAGCCAATTCCAATACAG |
| 116 | Tdh2t-AtCPR1-R | | AAGTAACTTAAGGAGTTAAATTTACCAGACGTCCCTCAAGTA |
| 117 | AtCPR1-Tdh2t-F | | TACTTGAGGGACGTCTGGTAAATTTAACTCCTTAAGTTACTT |
| 118 | Tdh2t-AtCPR2-R | | AAGTAACTTAAGGAGTTAAATTTACCAGACGTCCCTCAAATA |
| 119 | AtCPR2-Tdh2t-F | | TATTTGAGGGACGTCTGGTAAATTTAACTCCTTAAGTTACTT |
| 120 | Tdh2t-TcCPR-R | | AAGTAACTTAAGGAGTTAAATTTACCAAATATCTCTCAAATA |
| 121 | TcCPR-Tdh2t-F | | TATTTGAGAGATATTTGGTAAATTTAACTCCTTAAGTTACTT |
| 122 | His3A-Tdh2t-R | | GCGCTCACCAAGCTCTTAAAACGCGAAAAGCCAATTAGTGTGA |
| 123 | Tdh2t-His3A-F | | TCACACTAATTGGCTTTTCGCGTTTTAAGAGCTTGGTGAGCGC |
| 124 | His3A-R | | GTCGTCTATGTGTAAGTCACCAATGC |
| ***CYP720B1* and *TcCPR* gene expression cassettes were integrated into δ site** | | | |
| 125 | δA-F | | TGTTGGAATAGAAATCAACTATCATCTACTAAC |
| 126 | His3-δA-R | | ACCAAGCTCTTAAAACGGGAAACAGGCGCTACCATGAGAAT |
| 127 | δA-His3-F | | ATTCTCATGGTAGCGCCTGTTTCCCGTTTTAAGAGCTTGGT |
| 128 | His3-pgk1p-F | | TTTCTCTTGAACTCGACGGATTATTTTAGATTCCTGACTTCAAC |
| 129 | pgk1p-His3-R | | GTTGAAGTCAGGAATCTAAAATAATCCGTCGAGTTCAAGAGAAA |
| 130 | Tdh2t-δB-F | | TCACACTAATTGGCTTTTCGCGCTTCGGTTACTTCTAAGGAA |
| 131 | δB-Tdh2t-R | | TTCCTTAGAAGTAACCGAAGCGCGAAAAGCCAATTAGTGTGA |
| 132 | δB-R | | GGTGAGGTTAACATTGGTGGTGGT |

**Table S2** Primers used for cassettes construction in this study

| **Strains** | **PCR fragments** | **Primers used for PCR fragments** | **Cassettes Constructed by  fusion PCR** | **Primers used Cassettes constuction** | **Parental strain** | | |
| --- | --- | --- | --- | --- | --- | --- | --- |
| W303-1a | none | none | none | none | - | | |
| WTI | *δ-Up* | 1,2 | *δ-Up* | 1,2 | W303-1a | | |
|  | *P_PGK1_* | 3,4 | *P_PGK1_- tHMG1-T_PGK1_* | 3,8 |  |  |  |
|  | *tHMG1* | 5,6 |  |  |  |  |  |
|  | *T_PGK1_* | 7,8 |  |  |  |  |  |
|  | *T_TDH3_* | 9,10 | *T_TDH3_-IDI1-T_ADH1_* | 9,10 |  |  |  |
|  | *IDI1* | 11,12 |  |  |  |  |  |
|  | *T_ADH1_* | 13,14 |  |  |  |  |  |
|  | *LEU2* | 15,16 | *LEU2-δ-Down* | 15,16 |  |  |  |
|  | *δ-Down* | 17,18 |  |  |  |  |  |
| WTI-BE | *δ-Up* | 19,20 | *δ-Up* | 19,20 | WTI | | |
|  | *P_PGK1_* | 21,22 | *P_PGK1_-BTS1-GGGS-ERG20- T_CYC1_* | 21,28 |  |  |  |
|  | *BTS1* | 23,24 |  |  |  |  |  |
|  | *ERG20* | 25,26 |  |  |  |  |  |
|  | *T_CYC1_* | 27,28 |  |  |  |  |  |
|  | *TRP1* | 29,30 | *TRP1-δ-Down* | 29,32 |  |  |  |
|  | *δ-Down* | 31,32 |  |  |  |  |  |
| W1 | *URA3-Up* | 39,40 | *URA3-Up* | 39,40 | WTI-BE | | |
|  | *P_TEF1_* | 41,42 | *P_TEF1_-LPS-T_ADH2_* | 41,52 |  |  |  |
|  | *LPS* | 43,50 |  |  |  |  |  |
|  | *T_ADH2_* | 51,52 |  |  |  |  |  |
|  | *URA3-Down* | 53,54 | *URA3-Down* | 53,54 |  |  |  |
| W2 | *LPS(M593I)* | 33,34 | *LPS^MM^; P_TEF1_-LPS^MM^-T_ADH2_* | 33,36;41,52 | WTI-BE | | |
|  | *LPSMM* | 35,38 |  |  |  |  |  |
|  | *LPS(Y700F)* | 37,36 |  |  |  |  |  |
| W3 | *P_TEF1_* | 41,44 | P_TEF1_- T40LPS-T_ADH2_ | 41,52 | WTI-BE | | |
|  | *T40LPS* | 45,50 |  |  |  |  |  |
| W4 | *T40LPS^MM^* | 45,50 | P_TEF1_- T40LPS^MM^-T_ADH2_ | 41,52 | WTI-BE | | |
| W5 | *P_TEF1_* | 41,46 | P_TEF1_- T60LPS-T_ADH2_ | 41,52 | WTI-BE | | |
|  | *T60LPS* | 47,50 |  |  |  |  |  |
| W6 | *T60LPS^MM^* | 47,50 | P_TEF1_- T60LPS^MM^-T_ADH2_ | 41,52 | WTI-BE | | |
| W7 | *P_TEF1_* | 48,46 | P_TEF1_- T79LPS-T_ADH2_ | 41,52 | WTI-BE | | |
|  | *T79LPS* | 49,50 |  |  |  |  |  |
| W8 | *T79LPS^MM^* | 48,50 | P_TEF1_- T79LPS^MM^-T_ADH2_ | 41,52 | WTI-BE | | |
| WTE | *ADE2-Up* | 55,56 | ADE2-Up | 55,56 | W303-1a | | |
|  | *P_PGK1_* | 57,58 | P_PGK1_-tHMG1-T_PGK1_ | 57,62 |  |  |  |
|  | *tHMG1* | 59,60 |  |  |  |  |  |
|  | *T_PGK1_* | 61,62 |  |  |  |  |  |
|  | *P_TDH3_* | 63,64 | P_TDH3_-ERG20-T_ERG20_ | 63,66 |  |  |  |
|  | *ERG20-T_ERG20_* | 65,66 |  |  |  |  |  |
|  | *LEU2* | 67,68 | LEU2- ADE2-Down | 67,69 |  |  |  |
|  | *ADE2-Down* | 69,70 |  |  |  |  |  |
| W9 | *9A* | 71,72 | 9A-ADE2 | 71,74 | WTE | | |
|  | *ADE2* | 73,74 |  |  |  |  |  |
|  | *PMET3* | 75,76 | PMET3-9B | 75,78 |  |  |  |
|  | *9B* | 77,78 |  |  |  |  |  |
| W10/W11/W12 | *URA3-Up* | 79,80 | URA3-Up | 79,80 | | WTI/WTE/W9 | |
|  | *P_TEF1_* | 81,82 | *P_TEF1_-T79LPS*(M593I-Y700F)*-T_ADH1_* | 81,86 | |  |  |
|  | *T79LPS^MM^* | 83,84 |  |  |  |  |  |
|  | *T_ADH1_* | 85,86 |  |  |  |  |  |
|  | *P_TEF1_* | 87,88 | *P_TEF1_-BTS1-T_ADH2_* | 87,92 | |  |  |
|  | *BTS1* | 89,90 |  |  |  |  |  |
|  | *T_ADH2_* | 91,92 |  |  |  |  |  |
|  | *URA3-Down* | 93,94 | URA3-Down | 93,94 | |  |  |
| WM | *rDNA-Up* | 95,96 | *rDNA-Up* | 95,96 | | | W9 |
|  | *P_TEF1_-T79LPS(M593I-Y700F)-T_ADH1_* | 97,86 | *P_TEF1_-T79LPS*(M593I-Y700F)*-T_ADH1_* | *97,86* | | |  |
|  | *P_TEF1_-BTS1-T_ADH2_* | 87,92 | *P_TEF1_-BTS1-T_ADH2_* | *87,92* | | |  |
|  | *URA3* | 98,93 | *URA3-rDNA-Down* |  | | |  |
|  | *rDNA-Down* | 99,100 |  |  |  |  |  |
| WM1 | *HIS3B* | 101,102 | *HIS3B* | 101,102 | | | WM |
|  | *P_PGK1_* | 103,104 | *P_PGK1_-CYP720B1-T_ADH1_* | 103,108 | | |  |
|  | *CYP720B1* | 105,106 |  |  |  |  |  |
|  | *T_ADH1_* | 107,108 |  |  |  |  |  |
|  | *P_TDH3_* | 109,110 | *P_TDH3_-AtCPR1-T_TDH2_* | 109,122 | | |  |
|  | *AtCPR1* | 111,116 |  |  |  |  |  |
|  | *T_TDH2_* | 117,122 |  |  |  |  |  |
|  | *HIS3A* | 123,124 | *HIS3A* | 123,124 | | |  |
| WM2 | *P_TDH3_* | 109,112 | *P_TDH3_-AtCPR2-T_TDH2_* | 109,122 | | | WM |
|  | *AtCPR2* | 113,118 |  |  |  |  |  |
|  | *T_TDH2_* | 119,122 |  |  |  |  |  |
| WM3 | *P_TDH3_* | 109,114 | *P_TDH3_-TcCPR-T_TDH2_* | 109,122 | | | WM |
|  | *TcCPR* | 115,120 |  |  |  |  |  |
|  | *T_TDH2_* | 121,122 |  |  |  |  |  |
| WMM | *δ-Up* | 125,126 | *δ-Up- HIS3* | 125,129 | | | WM |
|  | *HIS3* | 127,129 |  |  |  |  |  |
|  | *P_PGK1_-CYP720B1-T_ADH1_* | 128,108 | *P_PGK1_-**CYP720B1-T_ADH1_* | 128,108 | | |  |
|  | *P_TDH3_-TcCPR-T_TDH2_* | 109,131 | *P_TDH3_-TcCPR-T_TDH2_* | 109,131 | | |  |
|  | *δ-Down* | 130,132 | *δ-Down* | 130,132 | | |  |


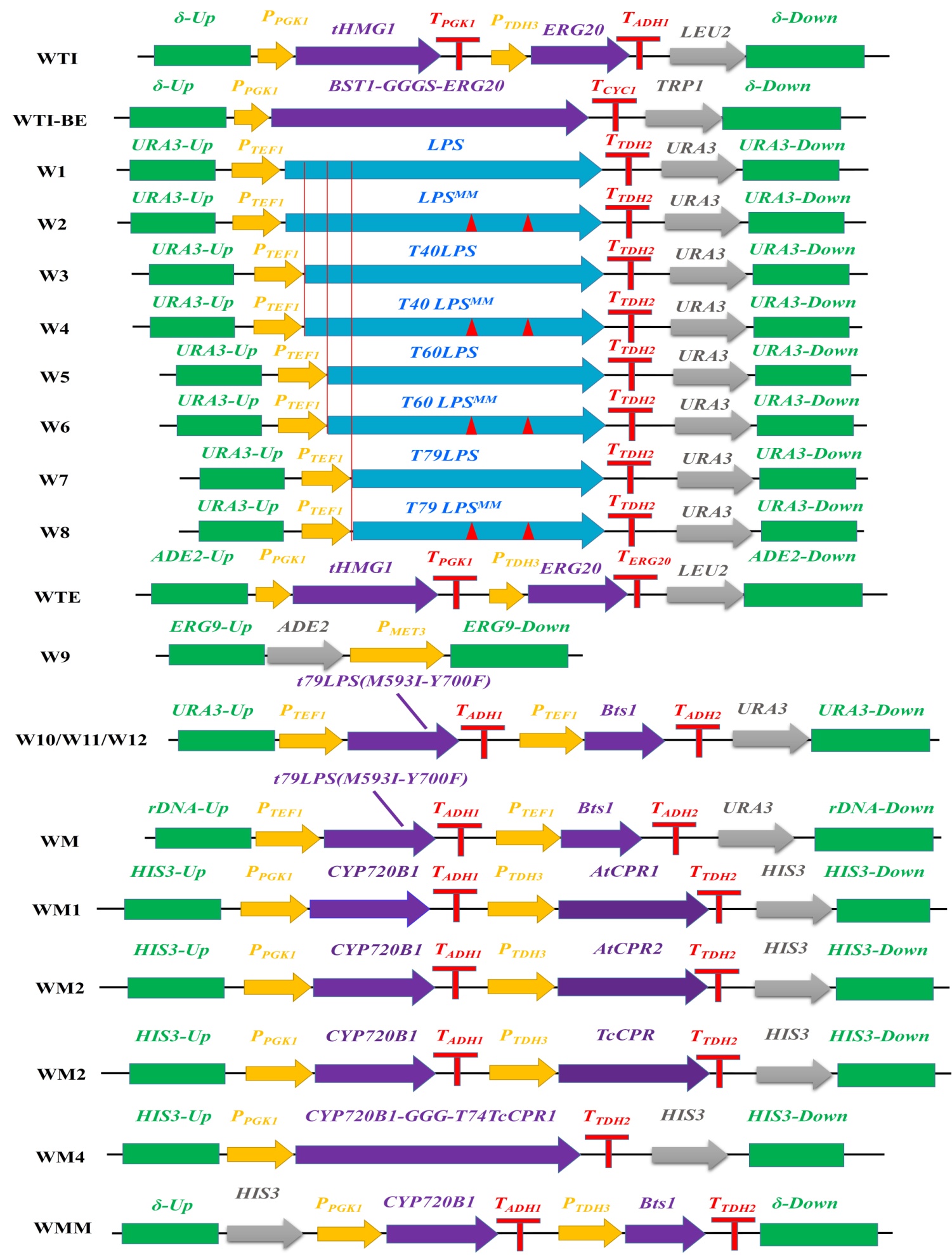


**Figure S1.** Construction of gene expression cassettes and yeast strains.
